# Harmonization of Multiple SARS-CoV-2 Reference Materials Using the WHO IS (NIBSC 20/136): Results and Implications

**DOI:** 10.3389/fmicb.2022.893801

**Published:** 2022-05-30

**Authors:** William Jonathan Windsor, Yannik Roell, Heidi Tucker, Chi-An Cheng, Sara Suliman, Laura J. Peek, Gary A. Pestano, William T. Lee, Heinz Zeichhardt, Molly M. Lamb, Martin Kammel, Hui Wang, Ross Kedl, Cody Rester, Thomas E. Morrison, Bennet J. Davenport, Kyle Carson, Jennifer Yates, Kelly Howard, Karen Kulas, David R. Walt, Aner Dafni, Daniel Taylor, May Chu

**Affiliations:** ^1^Colorado School of Public Health, Center for Global Health, Aurora, CO, United States; ^2^Division of Infectious Diseases, New York State Department of Health, Wadsworth Center, Albany, NY, United States; ^3^Department of Pathology, Brigham and Women's Hospital, Boston, MA, United States; ^4^Division of Rheumatology, Inflammation and Immunity, Harvard Medical School, Brigham and Women's Hospital, Boston, MA, United States; ^5^Division of Experimental Medicine, Zuckerberg San Francisco General Hospital, University of California, San Francisco, San Francisco, CA, United States; ^6^Biodesix, Inc., Boulder, CO, United States; ^7^INSTAND e.V., Society for Promoting Quality Assurance in Medical Laboratories, Duesseldorf, Germany; ^8^IQVD GmbH, Institut fuer Qualitaetssicherung in der Virusdiagnostik, Berlin, Germany; ^9^GBD Gesellschaft fuer Biotechnologische Diagnostik mbH, Berlin, Germany; ^10^Thermo Fisher Scientific, Waltham, MA, United States; ^11^Department of Immunology, University of Colorado Anschutz Medical Campus, Aurora, CO, United States; ^12^Oneworld Accuracy, Vancouver, BC, Canada

**Keywords:** SARS-CoV-2, serology, International Standards, concordance, immunology, harmonization, parallel-line assay

## Abstract

**Background:**

There is an urgent need for harmonization between severe acute respiratory syndrome coronavirus 2 (SARS-CoV-2) serology platforms and assays prior to defining appropriate correlates of protection and as well inform the development of new rapid diagnostic tests that can be used for serosurveillance as new variants of concern (VOC) emerge. We compared multiple SARS-CoV-2 serology reference materials to the WHO International Standard (WHO IS) to determine their utility as secondary standards, using an international network of laboratories with high-throughput quantitative serology assays. This enabled the comparison of quantitative results between multiple serology platforms.

**Methods:**

Between April and December 2020, 13 well-characterized and validated SARS-CoV-2 serology reference materials were recruited from six different providers to qualify as secondary standards to the WHO IS. All the samples were tested in parallel with the National Institute for Biological Standards and Control (NIBSC) 20/136 and parallel-line assays were used to calculate the relevant potency and binding antibody units.

**Results:**

All the samples saw varying levels of concordance between diagnostic methods at specific antigen–antibody combinations. Seven of the 12 candidate materials had high concordance for the spike-immunoglobulin G (IgG) analyte [percent coefficient of variation (%CV) between 5 and 44%].

**Conclusion:**

Despite some concordance between laboratories, qualification of secondary materials to the WHO IS using arbitrary international units or binding antibody units per milliliter (BAU/ml) does not provide any benefit to the reference materials overall, due to the lack of consistent agreeable international unit (IU) or BAU/ml conversions between laboratories. Secondary standards should be qualified to well-characterized reference materials, such as the WHO IS, using serology assays that are similar to the ones used for the original characterization of the WHO IS.

## Introduction

There is an urgent need for harmonization between severe acute respiratory syndrome coronavirus 2 (SARS-CoV-2) serology platforms and assays prior to defining appropriate correlates of protection and as well inform the development of new rapid diagnostic tests that can be used for serosurveillance as new variants of concern (VOC) emerge (Berry et al., 2020; Ciotti et al., 2021; Giavarina and Carta, 2021; Infantino et al., 2021; Perkmann et al., 2021; Petrone et al., 2021; Knezevic et al., 2022).

Conversion of results from different laboratory methods to a harmonized international unit reduces the interlaboratory/method variability (Cooper et al., 2018; McDonald et al., 2018; Mattiuzzo et al., 2019, 2020; Ciotti et al., 2021; Knezevic et al., 2022). The WHO International Standards (ISs) are considered the highest quality materials to use for comparison between diagnostic methods using international units (Mattiuzzo et al., 2020). The WHO IS for SARS-CoV-2 serology standard is the National Institute for Biological Standards and Control (NIBSC) 20/136 (United Kingdom, 2020). This standard, as most biological standards, was produced in limited quantities, making it difficult to be used exclusively as a calibrant to compare results between multiple SARS-CoV-2 serology assays on a global scale. Therefore, there is a pressing need to increase the availability of appropriate reference materials that are considered equivalent to the WHO IS. Other well-characterized reference samples can be evaluated against the WHO IS to obtain a valid measurement and calibrated to the arbitrary WHO IS values of 1,000 international units per milliliter (IU/ml) for neutralization assays and 1,000 binding antibody units per milliliter (BAU/ml) (National Institute for Biological Standards Control, 2020).

We compared multiple SARS-CoV-2 serology reference materials to the WHO IS to determine their utility as secondary standards, using an international network of laboratories with high-throughput quantitative serology assays. This enabled the comparison of quantitative results between multiple serology platforms. Furthermore, each serology method can derive a BAU/ml (or IU/ml as appropriate) conversion for multiple antigen–antibody combinations within each sample that are scaled to the arbitrary 1,000 BAU/ml value assigned to the WHO IS. We also note that neutralization assays that report IU/ml may additionally be calibrated to the WHO IS.

## Materials and Methods

### Recruitment of Severe Acute Respiratory Syndrome Coronavirus 2 Serology Reference Materials

Between April and December 2020, 13 well-characterized and validated SARS-CoV-2 serology reference materials were recruited from six different providers (Table 1) (National Institute for Biological Standards Control, 2020; Frederick National Laboratory for Cancer Research, 2021; Oneworld Accuracy, 2021; Thermo Fisher Scientific, 2021; Windsor et al., 2021; Zeichhardt and Kammel, 2021). Reference materials were selected based on the following criteria: originally characterized by the suppliers with the relevant test's thresholds for positive and negative results, are readily available, enough panels will exist after this study to distribute for widespread use, and the providers intend to distribute their reference materials to other (primarily low-resource) laboratories. All the materials were individually evaluated against the WHO IS using previously validated diagnostic tests given in Table 2 and characterized according to the anticipated results shown in Table 1. All the reference materials and diagnostic tests were handled according to manufacturers' and the respective Clinical Laboratory Improvement Amendments (CLIA) laboratory developed test instructions.

**Table 1:** Severe acute respiratory syndrome coronavirus 2 (SARS-CoV-2) serology harmonization reference material providers.

**Institution**	**Type of provider**	**SARS-CoV-2 serology panel name**	**Sample IDs**	**Material type**	**Anticipated results from development**
University of Colorado	Academic/Research	COVID-19 Serology Control Panel (Windsor et al., 2021)	CSCP-HR	Pooled Convalescent Plasma	N-IgG, Total = Reactive; RBD-IgG, Total = Highly Reactive; S-IgG, Total = Reactive
			CSCP-WR	Pooled Convalescent Plasma, 1:4 dilution of the CSCP_HR	N-IgG, Total = Reactive; RBD-IgG, Total = Reactive; S-IgG, Total = Reactive
			CSCP-NR	Pre-2019 Donor Plasma	Non-Reactive
NCI Frederick Lab	Government	Human SARS-COV-2 Serology Standard (Frederick National Laboratory for Cancer Research, n.d.)	NCI Frederick	Pooled Convalescent Plasma	N-IgG = Reactive; N-IgM = Reactive; S-IgG = Reactive; S-IgM = Reactive
Oneworld Accuracy	Commercial	COVS434 | SARS-CoV-2 Serology (Oneworld Accuracy, 2021)	1WA-A	Single Donor Human Plasma	No Ag indication, IgG against SARS-CoV-2, Total = Reactive
			1WA-B	Single Donor Human Plasma	No Ag indication, IgG against SARS-CoV-2, Total = Reactive
			1WA-C	Single Donor Human Plasma	No Ag indication, IgG+IgM against SARS-CoV-2, Total = Reactive
			1WA-D	Pre-2019 Donor Plasma	Non-Reactive
INSTAND	Commercial	Samples from EQA scheme (416) SARS-CoV-2 (Ak) (Zeichhardt and Kammel, n.d.)	416006	Convalescent Serum of a single donor after infection with human coronaviruses OC43 and HKU1 (single donation, blood collected 2 years after last infection)	Non-Reactive
			416029	Convalescent Serum of a donor after SARS-CoV-2 infection (single donation, blood collected 154 day after onset of disease)	N-IgG, Total = Reactive; RBD/S-IgG, Total = Reactive
			416048	Post Pfizer-BioNTech COVID-19 Vaccine donor Serum (single donation, blood collected 63 days after 2^nd^ vaccination; no prior evidence of infection)	N-IgG, Total = Non-Reactive; RBD/S-IgG, Total = Reactive
Thermo Fisher	Commercial	MAS™ SARS-CoV-2 IgG Positive Control Kit (Cat# 10028305) (Thermo Fisher Scientific, n.d.)	ThermoFisher	Pooled COVID-19 positive human plasma added to difibrinated plasma with ProClin 950 and Sodium azide	N-IgG,Total = Reactive; RBD-IgG, Total = Reactive; S-IgG, Total = Reactive
National Institute for Biological Standards and Controls	Government	NIBSC 20/136 (National Institute for Biological Standards Control, 2020)	WHO IS	Pooled Convalescent Plasma	1000 BAU/mL for IgM, IgG, and IgA subtypes

**Table 2:** SARS-CoV-2 serology harmonization testing laboratories and methods.

**Institution**	**Type of lab**	**Platform**	**Method**	**Antigen targets**	**Antibodies**
University of Colorado	Academic/ Research	Lab-Developed Test	SARS-CoV Focus Reduction Neutralization Titer (FRNT)	2019 n-CoV/USA-WA1/2020	Total Ig
Biodesix, Inc.	Commercial	GenScript cPass Nab	Neutralization(Nab) ELISA	RBD	Total Ig
		Bio-Rad Platelia	ELISA	N	IgG, IgM, IgA
Brigham and Women's Hospital	Academic/ Clinical	Laboratory Developed Test upon Quanterix Simoa HD-X platform	Multiplexed Single Molecule Array (MSMA)	S, RBD, N, S1	IgG, IgM, IgA
Wadsworth Center, New York State Department of Health	Reference/ Public Health	Lab-Developed Test upon Luminex Platform	Multiplexed microsphere assay (MMA)	S, RBD, N, S1, S2	IgG, IgM, IgA, Total Ig
University of Colorado	Academic/ Research	Lab-Developed Test	Multiplex microsphere immunoarray (MIA)	N, RBD, S1, S2	IgG

### Neutralization Assays

#### Severe Acute Respiratory Syndrome Coronavirus 2 Focus Reduction Neutralization Test

Vero E6 cells (ATCC, CRL-1586; Manassas, Virginia, USA) were maintained at 37°C in Dulbecco's Modified Eagle Medium (DMEM) (HyClone 11965-084; Logan, Utah, USA) supplemented with 10% fetal bovine serum and 100 U/ml penicillin-streptomycin. SARS-CoV-2 strain 2019 n-CoV/USA-WA1/2020 was obtained from ATCC. The virus was passaged once in Vero E6 cells and titrated by the focus reduction neutralization test (FRNT) on Vero E6 cells. All the work with infectious SARS-CoV-2 was performed in Biosafety Level 3 (BSL3) facilities at the University of Colorado School of Medicine.

The focus reduction neutralization test (FRNT) was performed as previously described (Annen et al., 2021; Schultz et al., 2021; Taylor et al., 2021). Vero E6 cells were seeded in 96-well plates at 10^4^ cells/well. On the next day, serum samples were heat inactivated at 56°C for 30 min and then serially diluted (2-fold, starting at 1:10) in DMEM supplemented with 1% fetal bovine serum (FBS) and 10 mM 4-(2-Hydroxyethyl)-1-piperazine ethanesulfonic acid (HEPES) (Merck, 7365-45-9, Darmstadt, Germany). Approximately, 100 focus-forming units (FFUs) of virus were added to each well and the serum/virus mixture was incubated for 1 h at 37°C. Following co-incubation of serum and virus, medium was removed from cells and the serum/virus mixture was added to the cells for 1 h at 37°C. Serum/virus mixture was removed and cells overlaid with 1% methylcellulose (MilliporeSigma, M0512; Burlington, Massachusetts, USA) in DMEM plus 2% FBS and incubated for 24 h at 37°C. Cells were fixed with 1% paraformaldehyde (PFA) (Acros Organics, 416780030; Morris Plains, New Jersey, USA) for 1 h, washed six times with phosphate-buffered saline-0.05% Tween 20 (PBS-T), and probed with 1 μg/ml of chimeric human anti-SARS-CoV spike antibody (CR3022, Absolute Antibody, Ab01680; Oxford, UK) in Perm Wash Buffer [1X PBS/0.1% saponin/0.1% bovine serum albumin (BSA)] for 2 h at 25°C. After three washes with PBS-T, cells were incubated with goat antihuman immunoglobulin G (IgG) Fc-horseradish peroxidase (HRP) (Southern Biotech, 2014-05; Birmingham, Alabama, USA) diluted at 1:1,000 in Perm Wash Buffer for 1.5 h at 25°C. SARS-CoV-2-positive foci were visualized with TrueBlue substrate (SeraCare, 5510-0030, Milford, Massachusetts, USA) and counted using the CTL BioSpot analyzer and BioSpot software (Cellular Technology Ltd., Shaker Heights, Ohio, USA). The FRNT50 titers were calculated relative to a virus only control (no serum) set at 100%, using GraphPad Prism 9.1.2 default nonlinear curve fit constrained between 0 and 100%.

#### CPass α-Receptor-Binding Domain (GenScript) Neutralization Antibody Test

The cPass α-receptor-binding domain (RBD) neutralization antibody (nAb) test is a quantitative assay that specifically measures a subset of spike-binding antibodies that can block the interaction between the RBD on the SARS-CoV-2 spike protein and the human host receptor angiotensin-converting enzyme 2 (ACE2) (GenScript, 2021). The assay is performed as a blocking ELISA as described in the Food and Drug Administration (FDA) Emergency Use Authorization (EUA) instructions for use in the cPass™ SARS-CoV-2 Neutralization Antibody Detection Kit. The surrogate virus neutralization test (SVNT) cPass assay was clinically validated and shown to be 100% sensitive and specific when compared to a gold standard plaque reduction neutralization test (PRNT), with qualitative analysis results 100% in agreement (GenScript, 2021). The reference materials were diluted and preincubated 1:1 with RBD protein conjugated to HRP at 37°C for 30 min. The mixture (100 μl) was then added to a 96-well plate coated with human ACE2 receptor protein; the plate was sealed and incubated for an additional 15 min at 37°C. The plate was washed four times with 260 μl/well Wash Solution provided in the kit before addition of 100 μl per well 3,3',5,5'-Tetramethylbenzidine (TMB) substrate for 15 min at room temperature. 50 μl of 1 N sulfuric acid solution was added to each well and the optical density (OD) was measured at 450 nm using a spectrophotometer. The nAb assay readout was percent signal inhibition by neutralizing antibodies, which was calculated to be the OD value of the sample relative to the OD of the negative control subtracted from one (Tan et al., 2020; Petrone et al., 2021; Taylor et al., 2021). The positive cutoff results are ≥ 30% signal inhibition and results <30% are reported negative based on previously conducted clinical validation studies (Petrone et al., 2021).

### Binding Antibody Assays

#### Platelia α-Nucleocapsid Total Antibody Test

The Platelia α-nucleocapsid (anti-N) total antibody test detects antibodies [IgG, immunoglobulin M (IgM), and immunoglobulin A (IgA) combined; Bio-Rad Incorporation] to the nucleocapsid protein. The assay is performed as a one-step antigen capture ELISA as described in the FDA EUA instructions for use for the Platelia SARS-CoV-2 Total Antibody Test Kit (Bio-Rad, 2021). The diluted plasma (1:5) and the WHO IS (1.5-fold serial dilution series up to 8 times, starting at 1:90 dilution) were mixed with SARS-CoV-2 nucleocapsid protein coupled with horseradish peroxidase (HRP) enzyme at a 1:1 ratio and 100 μl added to a 96-well plate coated with the nucleocapsid protein. The plate was covered with an adhesive plate sealer and incubated at 37°C for 1 h. The plate was then washed five times with the Working Washing Solution provided in the kit and 200 μl of the Enzyme Development Solution was added to each well. After a 30-min incubation in the dark at room temperature (18–30°C), the reaction was stopped by adding 100 μl per well of an acidic stopping solution and mixing thoroughly before measuring the OD at 450 nm using a spectrophotometer. The assay readout was a ratio of the specimen OD to cutoff control OD. A positive specimen-to-cutoff ratio ≥ 1.0 and <0.8 is negative and in between is reported equivocal with the recommendation of another specimen collected 3 days later. The Platelia assay has FDA EUA clearance for a qualitative interpretation of results (Bio-Rad, 2021).

#### Simoa Serology Assay

Simoa assays for IgG, IgA, and IgM against four SARS-CoV-2 targets (spike, S1, nucleocapsid, and RBD) were performed as previously described (Norman et al., 2020). Reference materials were diluted 1:250-, 1:1,000-, 1:4,000-, and 1:16,000-fold in Homebrew Detector/Sample Diluent (Quanterix Corporation, Product code: 101359, Billerica, Massachusetts, USA). Four antigen-conjugated capture beads were mixed and diluted in Bead Diluent (Quanterix Corporation, Product code: 101362, Billerica, Massachusetts, USA), with a total of 500,000 beads per reaction (125,000 of each bead type). Biotinylated antibodies were diluted in Homebrew Detector/Sample Diluent to final concentrations of IgG (Bethyl Laboratories A80-148B; Montgomery, Texas, USA): 7.73 ng/ml, IgA (Abcam ab214003, Waltham, Massachusetts, USA): 150 ng/ml, and IgM (Thermo Fisher Scientific, MII0401, Pittsburgh, Pennsylvania, USA): 216 ng/ml: Streptavidin-β-galactosidase (SβG) concentrate (Quanterix Corporation, Product code: 1013397, Billerica, Massachusetts, USA) was diluted to 30 pM in SβG Diluent (Quanterix Corporation, Product code: 100376, Billerica, Massachusetts, USA). The serology assay was performed on the HD-X Analyzer (Quanterix) in an automated three-step assay. Average enzymes per bead (AEB) values were calculated by the HD-X Analyzer software (Norman et al., 2020).

#### Multiplexed Microsphere Assay

Specimens were assessed for the presence of antibodies reactive with SARS-CoV-2 using a multiplexed microsphere assay (MMA). Recombinant SARS-CoV-2 full-length spike, nucleocapsid, S2 (The Native Antigen Company, REC31868, REC31812, and REC31807, respectively, Kidlington, Oxfordshire, UK), RBD, and S1 (Mass Biologics, https://www.umassmed.edu/massbiologics, Boston, Massachusetts, USA) subunits were covalently linked to the surface of fluorescent microspheres (Luminex Corporation, LC10047, LC10006, LC10071, LC10061, and LC10023, respectively, Austin, Texas, USA). Serum samples (25 μl at doubling dilutions from 1:50 to 1:102,400) and antigen-coupled microspheres (25 μl at 5 × 104 microspheres/ml) were mixed and incubated 30 min at 37°C. Serum-bound microspheres were washed and incubated with phycoerythrin (PE)-conjugated secondary antibody. The PE-conjugated antibodies were chosen to specifically recognize total Ig (Pan-Ig), IgM, IgA, and IgG (Southern Biotechnology Associates Incorporation, 2010–2009, 2020–2009, 2050–2009, and 2040–2009, respectively, Birmingham, Alabama, USA). After washing and final resuspension in buffer, the samples were analyzed on the FlexMap 3D analyzer using xPONENT software (Luminex Corporation, version 4.3, Austin, Texas, USA).

### Multiplexed Microsphere Immunoassay (MIA)

A multiplexed microsphere immunoassay (MIA) was developed using BioLegend carboxylated LEGENDplex microbeads to simultaneously quantify IgG and IgA against the spike RBD and nucleocapsid of the Wuhan strain of SARS-CoV-2 (BEIresources.org, NR-52366, North Bethesda, Maryland, USA), three variants of concern beta gamma, delta (BEIresources.org, NR-54004/54005, North Bethesda, Maryland, USA), three season coronavirus strains (OC43, 229E, and HKU1) (BEIresources.org, NR-53713, North Bethesda, Maryland, USA), and tetanus toxoid (TT) (MilliporeSigma, #582231-25UG, St. Louis, Mosby, USA) as a positive control. Bovine serum albumin (BSA) (10%) (MilliporeSigma, #A7030, St. Louis, Mosby, USA) conjugated beads were used as a negative control. Multiplex bead protein conjugation, sample incubation, and flow cytometric analysis were performed as previously described (Schultz et al., 2021). Geometric mean fluorescence intensity (gMFI) of the IgG/IgA for each sample and dilution was captured with the CytoFLEX S Flow Cytometer (Beckman Coulter, Indianapolis, Indiana, USA) and analyzed with FlowJo (version 10.7.1; BD Biosciences, San Jose, California, USA). Prism (version 8.4.3, GraphPad) was used to plot data (Schultz et al., 2021).

### Statistical Analysis

Parallel-line assay (PLA) was used to compare all the secondary standard candidate samples to the WHO IS; all the analytes were set at 1,000 IU or BAU/ml (Finney and Schild, 1966). All the samples were tested in triplicate with each diagnostic test at dilutions within each assay's given linear range for the WHO IS. Data were analyzed using PLA analysis using R 3.5.0 that we created (R Core Team, 2021). Sample results and their corresponding dilutions were log-transformed and assessed for parallelism using the relative slope calculated individually between each sample and the WHO IS. To ensure the assumption of parallelism for PLA analysis to occur, a relative slope between 0.8 and 1.2 was considered parallel and samples with relative slopes outside the range were excluded from further analysis because they violated the PLA assumption of parallel lines (Mattiuzzo et al., 2020). The relative potency was calculated for each sample whose slope was within 20% of the WHO IS slope. Relative potencies were then converted to IU or BAU/ml based on the assay used (Finney and Schild, 1966) and parametric bootstrapping was used to calculate CIs for each sample (B. Efron, 1979; Landes et al., 2019). The full reproducible code and readme file are both available at: github.com/yroell/pla. and the overview of our created PLA analysis is shown in Supplementary Figure 1 showing an overview used for each sample. IU and BAU/ml conversions were then compared for interassay variability using percent coefficient of variation (%CV) (Reed et al., 2002; Wood et al., 2012).

## Results

### Analysis of Samples and Binding Antibody Unit Conversions

Thirteen samples (including the WHO IS) from six different providers (Table 1) were tested using six different SARS-CoV-2 serology diagnostic platforms. Twenty-one total antigen–antibody (Ag–Ab) combinations were evaluated. Three of the platforms were multiplexed platforms targeting multiple Ag–Ab combinations. The remaining three platforms consisted of two SARS-CoV-2 neutralization tests and one nucleocapsid-specific ELISA (Table 2). Each laboratory performed serial dilutions of the WHO IS to establish the linear range of the WHO within each testing platform. All the reference samples were then serial diluted within the WHO IS linear range and tested in triplicate.

**Figure 1:**
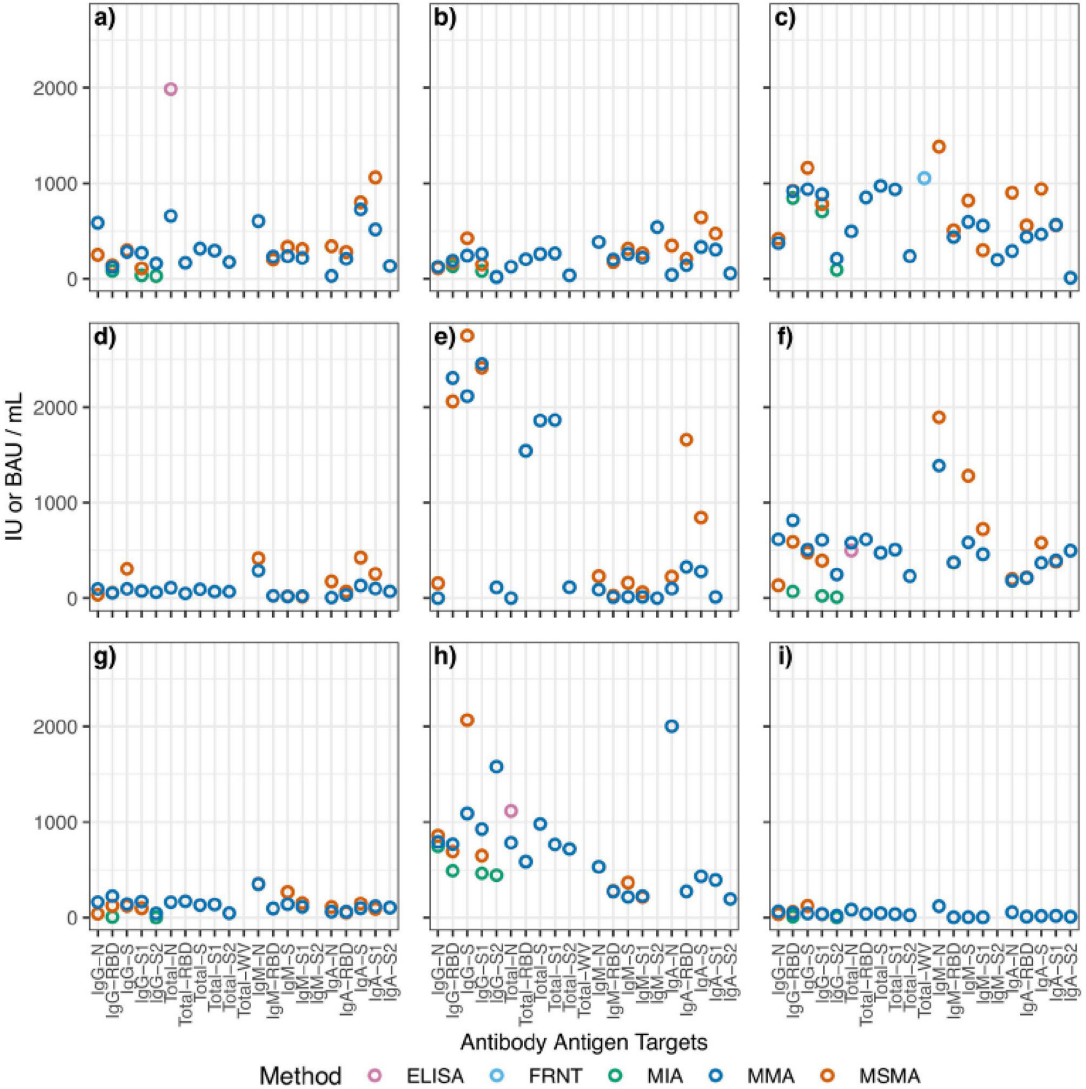
Aggregated scatterplot of computed binding antibody unit conversions for each reference sample. The following samples are represented by each subfigure: **(a)** 1WA-A; **(b)** 1WA-B; **(c)** 1WA-C; **(d)** 416029; **(e)** 416048; **(f)** CSCP-HR; **(g)** CSCP-WR; **(h)** NCI Frederick; **(i)** ThermoFisher. MIA, multiplexed microsphere immunoarray; MMA, multiplexed microsphere assay; Nab, neutralization; ELISA, enzyme-linked immunosorbent assay; FRNT, focus-reduction neutralization titer. The following samples were removed because they were classified as “non-reactive” during testing: 1WA-D, 416006, CSCP-NR.

Results from each laboratory were compiled and evaluated using PLA. Reference material samples were considered “parallel” if their relative slope against the WHO IS was between 0.8 and 1.2. Samples that failed to fall within the range were excluded from further analysis. For each sample at each Ag–Ab combination, BAUs (or IUs for neutralization tests) were calculated using sample relative potency. BAU conversions for each sample are shown in Table 3 and Figure 1 summarizes the BAU or IU conversions for each sample at each analyte. The IgG-spike analyte had more consistent BAU or IU/ml conversions between methods, regardless of the sample type. For example, the multiplexed microsphere assay (MMA) and MSMA results for the oneworld accuracy a(b,c,d) (1WA-A) sample were 281 (95% CI = 280–282) and 299 (95% CI = 294–304) BAU/ml, respectively; for the 1WA-C sample, the MMA and MSMA results were 938 (95% CI = 936–940) and 1,150 (95% CI = 1,142–1,178) BAU/ml, respectively. Further, for the covid-19 serology control panel-high reactive (CSCP-HR) sample, the MMA and MSMA results were 507 (95% CI = 506–508) and 477 (95% CI = 469–485) BAU/ml, respectively, and for the covid-19 serology control panel-weak reactive (CSCP-WR) sample, the MMA and MSMA results were 141 (95% CI = 0) and 120 (95% CI = 118–122) BAU/ml, respectively. Oppositely, the IgG-nuclecapsid analytes saw wider differences in BAU or IU/ml conversions between methods within the CSCP-HR, covid-19 serology control panel-non reactive (CSCP-NR), Thermo Fisher Scientific, 416,026, and 416,048 samples.

**Table 3:** Binding antibody unit conversions for serology harmonization samples.

			**416006**		**416029**		**416048**		**1WA–A**		**1WA–B**		**1WA–C**		**1WA–D**		**CSCP–HR**		**CSCP–NR**		**CSCP–WR**		**NCI Frederick**		**Thermo Fisher**	
**Ab**	**Ag**	**Method**	**BAU**	**95% CI**	**BAU**	**95% CI**	**BAU**	**95% CI**	**BAU**	**95% CI**	**BAU**	**95% CI**	**BAU**	**95% CI**	**BAU**	**95% CI**	**BAU**	**95% CI**	**BAU**	**95% CI**	**BAU**	**95% CI**	**BAU**	**95% CI**	**BAU**	**95% CI**
**Total Ig**	**N**	ELISA	NA	NA	NA	NA	NA	NA	1986	1958–2014	NA	NA	NA	NA	NA	NA	497	594–500	NA	NA	NA	NA	1116	1113–1119	NA	NA
		MMA	NA	NA	109	0	0	0	658	656–660	126	125–127	496	495–497	NA	NA	579	577–581	NA	NA	160	159–161	783	781–785	82	0
	**RBD**	MMA	NA	NA	49	0	1542	1,538–1,548	167	0	204	203–205	851	849–853	NA	NA	615	613–617	NA	NA	170	169–171	585	583–587	39	0
		Nab	NA	NA	NA	NA	NA	NA	NA	NA	NA	NA	NA	NA	NA	NA	NA	NA	NA	NA	NA	NA	NA	NA	NA	NA
	**S**	MMA	NA	NA	93	0	1860	1,854–1,866	315	314–316	260	259–261	972	969–975	NA	NA	474	473–475	NA	NA	129	0	980	977–983	45	0
	**S1**	MMA	NA	NA	68	0	1865	1,859–1,871	294	293–295	266	265–267	936	934–938	NA	NA	507	506–508	NA	NA	136	0	765	763–767	36	0
	**S2**	MMA	NA	NA	68	0	115	0	175	0	36	0	237	236–238	NA	NA	230	229–231	NA	NA	46	0	718	716–720	25	0
	**WV**	FRNT	NA	NA	NA	NA	NA	NA	NA	NA	NA	NA	1054	1045–1063	NA	NA	NA	NA	NA	NA	NA	NA	NA	NA	NA	NA
**IgG**	**N**	MIA	NA	NA	NA	NA	NA	NA	NA	NA	NA	NA	NA	NA	NA	NA	NA	NA	NA	NA	NA	NA	745	723–767	NA	NA
		MMA	NA	NA	96	0	0	0	585	583–587	125	124–126	373	372–374	NA	NA	616	614–618	NA	NA	161	160–162	792	2	62	0
		MSMA	NA	NA	35	34–36	156	149–173	251	248–254	110	106–114	419	406–432	NA	NA	135	132–138	NA	NA	40	39–41	856	843–869	36	35–37
	**RBD**	MIA	NA	NA	NA	NA	NA	NA	82	79–85	129	126–132	846	832–860	0	0	69	67–71	NA	NA	7	0	489	480–498	11	12–Oct
		MMA	NA	NA	54	0	2306	2,294–2,316	124	0	189	188–190	917	914–920	NA	NA	815	813–817	NA	NA	224	223–225	768	766–770	48	0
		MSMA	NA	NA	58	56–60	2060	2,020–2,100	142	139–145	167	160–174	924	905–943	NA	NA	589	576–602	NA	NA	123	119–127	691	676–706	58	56–60
	**S**	MMA	NA	NA	97	0	2115	2,109–2,121	281	280–282	243	242–244	938	936–940	NA	NA	507	506–508	NA	NA	141	0	1090	1088–1092	43	0
		MSMA	NA	NA	307	301–313	2749	2,703–2,792	299	294–304	424	415–433	1160	1,142–1,178	NA	NA	477	469–485	NA	NA	120	118–122	2067	2032–2102	120	118–122
	**S1**	MIA	NA	NA	NA	NA	NA	NA	36	31–41	81	75–87	703	781–725	NA	NA	24	23–25	NA	NA	NA	NA	463	448–478	NA	NA
		MMA	NA	NA	74	0	2453	2,441–2,465	271	270–272	260	259–261	883	881–885	NA	NA	609	608–610	NA	NA	167	0	925	923–927	38	0
		MSMA	NA	NA	75	73–77	2411	2,373–2,450	108	106–110	149	146–152	783	770–796	NA	NA	393	386–400	NA	NA	97	95–99	647	636–658	35	34–36
	**S2**	MIA	NA	NA	NA	NA	NA	NA	29	27–31	20	19–21	92	90–94	NA	NA	10	0	NA	NA	1	0	443	436–450	1	0
		MMA	NA	NA	61	0	113	0	160	159–161	18	0	210	0	NA	NA	247	246–248	NA	NA	43	0	1580	1576–1584	20	0
**IgM**	**N**	MMA	NA	NA	288	287–289	89	0	603	601–605	384	382–386	8360	8336–8384	NA	NA	1387	1381–1393	NA	NA	349	349–351	531	529–533	118	117–119
		MSMA	120	112–128	417	497–437	228	211–245	NA	NA	NA	NA	1383	1,314–1,452	18	16–20	1894	1702–2086	24	22–26	352	325–379	NA	NA	NA	NA
	**RBD**	MMA	NA	NA	25	0	10	0	232	231–233	202	0	436	435–437	NA	NA	375	374–376	NA	NA	94	0	273	272–274	3	0
		MSMA	6	0	24	0	27	0	200	198–202	174	172–176	507	503–511	4	0	372	368–376	NA	NA	94	92–96	279	276–282	NA	NA
	**S**	MMA	NA	NA	17	0	13	0	236	235–237	261	0	595	594–596	NA	NA	583	582–584	NA	NA	139	0	215	214–216	4	0
		MSMA	13	0	21	20–22	160	158–162	333	330–336	313	308–318	819	811–827	NA	NA	1280	1,268–1,292	NA	NA	267	264–270	365	362–368	NA	NA
	**S1**	MMA	NA	NA	22	0	13	0	217	216–218	221	220–222	556	555–557	NA	NA	459	458–460	NA	NA	111	0	224	223–225	3	0
		MSMA	4	0	14	13–15	63	61–65	310	307–313	266	262–270	299	296–302	NA	NA	723	716–730	NA	NA	152	151–153	213	210–216	NA	NA
	**S2**	MMA	NA	NA	NA	NA	NA	NA	NA	NA	541	533–549	198	187–209	NA	NA	NA	NA	NA	NA	NA	NA	NA	NA	NA	NA
**IgA**	**N**	MMA	NA	NA	6	0	0	0	30	29–31	41	40–42	290	289–291	NA	NA	182	181–183	NA	NA	60	0	2003	1978–2028	55	0
		MSMA	NA	NA	175	171–179	NA	NA	340	335–345	347	338–356	900	891–909	NA	NA	200	196–204	74	72–76	109	107–111	NA	NA	NA	NA
	**RBD**	MMA	NA	NA	35	0	99	0	209	208–210	141	0	437	436–438	NA	NA	211	210–212	NA	NA	60	0	274	273–275	10	0
		MSMA	2	0	69	68–70	224	222–226	279	277–281	211	210–212	557	552–562	1	0	216	214–218	0	0	51	49–53	NA	NA	NA	NA
	**S**	MMA	NA	NA	133	132–134	326	324–328	726	723–729	332	331–333	465	463–467	NA	NA	369	367–370	NA	NA	97	0	432	430–434	17	0
		MSMA	37	36–38	425	422–428	1659	1646–1672	800	794–806	642	638–646	941	933–949	10	0	579	574–584	NA	NA	146	143–149	NA	NA	NA	NA
	**S1**	MMA	NA	NA	100	99–101	279	277–281	517	515–519	305	304–306	564	561–567	NA	NA	394	391–397	NA	NA	117	116–118	392	389–395	18	0
		MSMA	NA	NA	254	252–256	844	837–851	1062	1053–1,071	473	470–476	559	554–564	NA	NA	382	379–385	NA	NA	90	88–92	NA	NA	NA	NA
	**S2**	MMA	NA	NA	70	69–71	12	0	134	133–135	56	0	10	0	NA	NA	496	493–499	NA	NA	103	102–104	194	192–196	9	0

Table 3 breaks down the computed BAU or IU/mL conversions and bootstrapped 95% Confidence Intervals of each method by their measured antibody and antigen analyte combination. ^*^NA indicates the result was either not measured by the particular assay, or the relative slope fell outside of 20% and therefore not possible to convert to International Units or Binding Antibody Units/mL (BAU); Ab, antibody; Ag, antigen; N, Nucleocapsid; S, Spike; RBD, Receptor Binding Domain; WV, Whole Virus; MSMA, multiplexed single molecule array; MIA, multiplexed microsphere immunoarray; MMA,multiplexed microsphere assay; Nab, neutralization; ELISA, enzyme–linked immunosorbent assay; FRNT, focus–reduction neutralization titer. A more comprehensive breakdown of the individual samples and test methods are found in (Tables 1, 2) respectively.

### Interlaboratory/Method Binding Antibody Units Concordance

**Figure 2:**
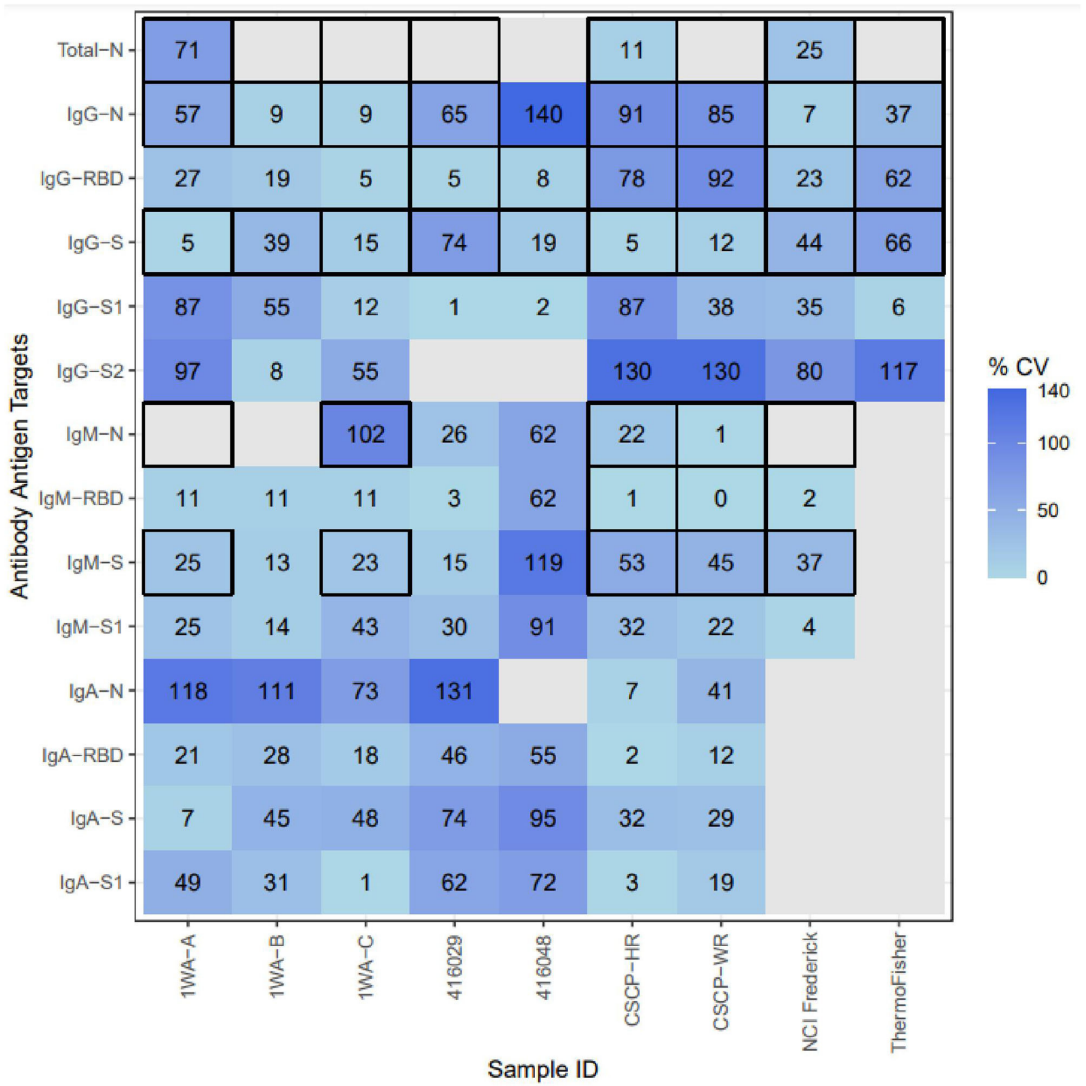
Inter-method concordance of binding antibody unit conversions among reference materials for each analyte. %CV, percent coefficient of variation; Light blue, Higher concordance between methods; dark blue, lower concordance between methods; blank, not enough labs yielded PLA results to compute concordance. Thick Black outlines indicate that the particular analyte was evaluated by that sample's provider. The following samples were removed because they were classified as “non-reactive” during testing: 1WA-D, 416006, CSCP-NR; The following Ag-Ab combinations were removed due to lack of sufficient PLA data due to linearity violations: Whole-Virus-Total, S2-Total, S2-IgM, S2-Iga, S2-Total, S-Total, RBD-Total.

Once BAUs were calculated, we evaluated results for overall intermethod concordance if multiple laboratories yielded results for each Ag–Ab combination using percent coefficient of variation (%CV). Lower %CV values (<21%) indicate that results are highly agreeable between laboratories. None of the samples tested yielded universally high concordance between methods (regardless of Ag–Ab combination). For specific Ag–Ab combinations, there was no universal concordance between methods regardless of the sample tested. Samples 1WA-A, 1WA-B, and 1WA-C saw high concordance between laboratories for both the IgG and IgM bound to S, RBD, and N antigens (%CV range between 5 and 57%). CSCP-HR and CSCP-WR were highly concordant within the IgG-S combination (5 and 12%, respectively) and IgA and IgM bound to S, RBD, and N antigens (%CV range between 2 and 53%). Sample 416,029 was highly concordant between laboratories for IgG-RBD and IgG-S1 combinations (%CV 6 and 1%, respectively). Sample 416,048 saw high concordance with IgG S, S1, and RBD combinations (%CV = 19, 2, and 8%, respectively). The highest %CV value in Figure 2 was found in sample 416,006 at the IgG-N analyte, which is likely because that particular sample was acquired from a postvaccinee individual and was not highly reactive to IgG-N during its characterization (Table 1). The National Cancer Institute (NCI) Frederick sample saw good overall concordance between laboratories for all the measured analytes. The IgG-S1 of the Thermo Fisher Scientific sample was highly concordant between laboratories (%CV = 6%). Result concordance between testing methods at each Ag–Ab combination is shown in Figure 2.

## Discussion

We evaluated multiple candidate reference materials against the WHO IS (NIBSC 20/136) to determine whether secondary standards could be established. We then evaluated the applicability of using arbitrary BAU conversions to compare results between laboratories and serology diagnostic methods. Many seroprevalence studies use different serology assays to estimate transmission and/or herd immunity. The differences between assays make it nearly impossible to harmonize and establish a reliable limit of detection. A reference standard would theoretically allow for comparison between such studies.

A number of studies have determined that internal standards provided by the WHO for various pathogens may be useful and should be used to compare results across laboratories and diagnostic methods to help establish correlates of protection for SARS-CoV-2 and other high-threat pathogens (Cooper et al., 2018; McDonald et al., 2018; Mattiuzzo et al., 2019, 2020; Ciotti et al., 2021; Knezevic et al., 2022). For example, when assessing candidate reference materials for enterovirus serology, one study evaluating the interassay variability for both the raw neutralization titer and the calculated relative potencies found a marked decrease in interassay variability. Their calculated percent geometric coefficient of variation (%GCV) was between 30 and 94% (Cooper et al., 2018), indicating that although their candidate materials had decreased interassay variability after the results were converted to a harmonized metric, it is difficult to know what is considered an acceptable coefficient of variation across methods in this context. Two additional studies that evaluated candidate reference materials for Zika virus found similar improvements to intermethod concordance with the reference material, yet GCVs remained exceptionally high, suggesting that a threshold for acceptable intermethod concordance may be difficult, if not impossible, to establish in these contexts (Mattiuzzo et al., 2019; Berry et al., 2020).

Finally, the developers of the WHO IS conducted a robust evaluation of the candidate standard that included 125 different SARS-CoV-2 serology assays (Mattiuzzo et al., 2020; Knezevic et al., 2022). When evaluating the interassay variability of results, they stratified their comparisons into neutralization assays, ELISAs, and “other” assays relative to what is now the WHO IS. Interassay variability between neutralization assays for samples tested relative to the WHO IS did not fall below 67% (%GCV range 67–250%). The interassay variability for the WHO IS itself was 241% (Mattiuzzo et al., 2020). Similar results were found when comparing ELISA methods and there were no data that evaluated the “other” methods included in the characterization. The assignment of an arbitrary 1,000 IU for neutralization assays and 1,000 BAU/ml for other assays—despite the large interassay variability relevant to the WHO IS—does not account for the vast differences between assays. Additionally, the interassay variability between all the methods used was not presented, which, therefore, makes it difficult to fully understand how best to harmonize results between multiple laboratories in order to assess correlates of protection. This study evaluated the interassay variability relative to the WHO IS across all the methods used. We also present the variability between laboratories for multiple Ag–Ab combinations to differentiate which ones are more likely to remain consistent or be highly variable within each sample.

Other studies also suggest that SARS-CoV-2 serology tests cannot be calibrated to the same measurement “ruler” and results compared between assays (Cooper et al., 2018; Bradley et al., 2021; Castillo-Olivares et al., 2021; Giavarina and Carta, 2021; Infantino et al., 2021; Perkmann et al., 2021; Solastie et al., 2021; Knezevic et al., 2022). It is also important to note that the IU or BAU assigned to the WHO IS is arbitrary and not based on an analytical concentration measurement. Additionally, results attained using the WHO IS are highly variable between assays. Our results demonstrate that any reference material should be characterized independently for each assay and it is not advisable to compare quantitative IU or BAUs between different assays. Therefore, arbitrary BAUs that were not calculated should not be used to benchmark any characterizations made for other reference materials, especially candidate secondary standards (Bradley et al., 2021; Giavarina and Carta, 2021; Perkmann et al., 2021). International Standards are not able to account for the wide variety of reagent formulations and nuances between testing methods using a universal metric such as an IU or BAU conversion. Finally, our findings show the qualification of secondary standards using the WHO IS using the 1,000 IU or BAU as a baseline metric that does not yield consistent IU or BAU conversions between assays.

Regardless of the pathogen, many other evaluations of “candidate” reference materials from the WHO have revealed a high degree of interassay and interlaboratory variability during characterization (Bozsoky, 1963; Holder et al., 1995; Wood et al., 2012; Dimech et al., 2013; Cooper et al., 2018; McDonald et al., 2018; Mattiuzzo et al., 2019; Kempster et al., 2020; Timiryasova et al., 2020). Although these findings cannot be verified within the context of this study, our findings reinforce that SARS-CoV-2 serology reference materials face the same challenges and interpretation issues that other groups have seen (Mattiuzzo et al., 2020; Castillo-Olivares et al., 2021; Ciotti et al., 2021; Giavarina and Carta, 2021; Infantino et al., 2021; Kristiansen et al., 2021). Standardization of IU or BAU values for candidate secondary standards relative to the WHO IS could not be achieved across different laboratory assays using methods consistent with the NIBSC characterization of the WHO IS (Mattiuzzo et al., 2020). This calls into question the feasibility of standardizing different serology assays in the future and what this means when interpreting seroprevalance or distinguishing between natural infections and vaccine-induced responses.

### Limitations

Some limitations are noted for this study. Among our laboratories, some were unable to yield relative potency values to use for a BAU/ml conversion for certain Ag–Ab combinations. Our criteria for PLA parallelism were more strict (relative slope = 0.8–1.2) than the standards set by the NIBSC (relative slope = 0.8–1.25) during the initial characterization of the NIBSC 20/136 because we wanted to set a more consistent range for relative slopes on either end (Mattiuzzo et al., 2020). Furthermore, the NIBSC does not clarify why they established an acceptable relative slope range of 0.8–1.25 was chosen. Manufacturing convalescent plasma/serum samples at scale is not common practice due to low volume donations and lot-to-lot differences. So, unlike molecular standards, it is difficult to generate large batches and consistent lots for harmonization or even for testing (in a postharmonization world). Two of the six methods used were neutralization assays; one did not yield relative potency for any samples tested and the other only yielded a relative potency for a single sample. Even after log, the raw candidate sample neutralization results failed to fall within the parameters to accurately perform PLA (Taylor et al., 2021).

Similar studies have used a variety of different interassay comparability methods that include, but are not limited to the Spearman's rank correlation coefficient, the Mann–Whitney *U* tests, and Bablok regression (McDonald et al., 2018; Castillo-Olivares et al., 2021; Giavarina and Carta, 2021; Perkmann et al., 2021). Percent coefficient of variation (%CV) is a flexible metric commonly used in clinical laboratories and the developers of International Standards to evaluate interassay, intralaboratory, and lot-to-lot variations (Reed et al., 2002; Mattiuzzo et al., 2020). Furthermore, each of the example of alternative comparison methods exclude outlier results from analysis, which biases comparisons to appear erroneously “better” in a study context where outlier laboratory results are important to consider when determining the effectiveness of candidate reference materials.

The MMA method tested the WHO standard as nonreactive (no reaction present) for IgM against the nucleocapsid and spike S2 and indeterminate (no result due to PLA violation) for IgA against the nucleocapsid. Even though the assay was sensitive enough to give values for these analytes, these numbers are below what was consider reactive. Because the standard was so low and set to 1,000 BAU/ml, any sample with detectable but similarly low quantities of an analyte will give a misleadingly high BAU/ml value and should be interpreted with caution.

Finally, each method in this study used different formulations of commercial reagents as noted in the Materials and Methods section. For coronavirus disease 2019 (COVID-19) and detection of anti-SARS-CoV-2 antibodies, the field is complicated by multiple antigen sources, multiple host experiences (one or more natural infections and/or vaccines and boosters), multiple variants, and multiple test platforms. This makes it very difficult to achieve harmony. The nuanced differences between these reagent, platforms, and host experiences might contribute to the differences between IU and BAU conversions. Serology is extremely dense with methods and tests, regardless of the pathogen, which highlights the difficulty of applying the same standards for interpretation because it does not account for the nuances that accompany a wide range of assays. This highlights the need for a more precise interpretation of reference material characterizations, so these differences can be accounted in future studies and allow for better harmonization of results between methods.

## Conclusion

Harmonization of serology reference materials will increase the accessibility of reference materials—particularly in low-resource settings, provided the methods used for comparison are accurate and reliable. Our findings indicate that the arbitrary units of the WHO IS are not an accurate means to compare SARS-CoV-2 serology results between different laboratories or methods. This study also shows that even after IU or BAU conversion, candidate secondary material results are still drastically different between laboratory methods. Both the International Standards and candidate secondary standards should only be used to compare the results within the same laboratory methods, provided they are using identical testing platforms, protocols, and reagent formulations (Bradley et al., 2021; Giavarina and Carta, 2021; Perkmann et al., 2021). This must be highlighted by regulatory bodies to accurately portray the use of the WHO IS as an assay calibrator during development or external quality assurance material for intramethod comparison, not as a universal comparator (Holder et al., 1995; Infantino et al., 2021).

Finally, despite some concordance between laboratories, qualification of secondary materials to the WHO IS using arbitrary IU or BAU/ml does not provide any benefit to the reference materials overall, due to the lack of consistent agreeable IU or BAU/ml conversions between laboratories. Secondary standards should be qualified to well-characterized reference materials, such as the WHO IS, using serology assays that are similar to the ones used for the original characterization of the WHO IS. However, secondary standards are useful if qualified using similar assays as the original characterization as source traceability for they can be used for intraassay adjustments and can be used in external quality assessment to identify binding to antigen(s) presented in an assay to a reference, thereby providing intralaboratory operations (Table 4).

**Table 4:** Recommendations for future development, use, and interpretation of International and Secondary Standards.

**Topic**	**Recommendation(s)**
Regulatory Bodies	•Replace the process that qualifies candidate secondary materials to an international standard with standards or best practices set for the “characterization” process of any potential reference materials using historical development of WHO IS' as a framework. *This will elevate the quality standards for characterization of samples.*
	•Regulatory bodies must also require more precise interpretation of how to use particular reference materials based on the results from their characterization. *These interpretations must take into account the nuances of reagent formulation, testing platform, and the results interpretation in a clinical setting. *
	•Once these interpretations are more precise, future studies can then appropriately compare the results between seroprevalance studies for SARS-CoV-2 and potentially other incoming pathogens of interest.
Reference Material Characterization	•When characterizing reference materials, the methodology, reagent formulation, and validation information must be shown and included in the interpretation of reference material testing results. Different assays with different reagent formulations might yield slightly different results.
	•Establish a minimum number of laboratory methods to include when characterizing potential reference materials.
	•Require that the development, manufacturing, and distribution of secondary standards align with Good Manufacturing Practices.
	•Establish a minimum list of pathogens to test for when determining sample microbial bioburden.
	•Establish a list of minimum requirements for “suitable assay” used to demonstrate reference material expected immunological activity.
	•Establish an acceptable level of concordance (%GCV or % CV) between laboratories for the average BAU IU conversion to be considered “reliable.”
Interpretation	•Clarify that reference material (international standards and secondary standards) characterization is extremely assay and context dependent, which can affect accuracy of result interpretations. Similar tests with similar reagents must be used when comparing BAU conversions, and seroprevalence study results.
	•Revoke the encouraged removal of outlier method results during sample characterization. Exclusion of outlier laboratory data that fall within the PLA assumptions makes reference materials less comparable between methods which might remove the ability to adequately compare results between seroprevalance studies.
	•In order to continue using any WHO IS after their supply runs out, consider the development artificial IS for serology.
	•Clarify and establish that the intended use of standard reference materials is for external quality assurance schemes, comparing results between studies using similar assays or reagents, and be used as “anchors” by testing the same standards in the beginning and the end of a longitudinal research study. Which will attest to the quality of the results presented by that research study.

## Data Availability Statement

The raw data supporting the conclusions of this article will be made available by the authors, without undue reservation.

## Author Contributions

WW oversaw the entire study operation, conducted the analyses, constructed the tables, and drafted the manuscript. YR conducted the data analyses in R, built an open source R script for the parallel-line assay (github.com/yroell/pla), and built all the figures (both the manuscript and supplementary). ML provided data analysis oversight. MC assisted WW with the overall study design, execution, and result interpretation. HT and WL provided their open source parallel-line assay analysis program to use as a comparison to the R script developed by YR. WW, HZ, MK, HW, AD, and DT contributed SARS-CoV-2 serology reference materials. The remaining authors tested the samples in their respective laboratorios. All authors contributed insights, edits, revisions, and interpretations to the final manuscript.

## Funding

This study is an expansion of the original COVID-19 Serology Control Panel Study funded by the Bill & Melinda Gates Foundation.

## Conflict of Interest

GP and LP was employed by Biodesix, Inc. HZ and MK was employed by INSTAND e.V., IQVD GmbH, and GBD Gesellschaft fuer Biotechnologische Diagnostik mbH. HW was employed by Thermo Fisher Scientific. AD and DT were employed by company Oneworld Accuracy. The authors declare that this study received funding from the Bill & Melinda Gates Foundation. The funder was not involved in the study design, collection, analysis, interpretation of data, the writing of this article or the decision to submit it for publication. The remaining authors declare that the research was conducted in the absence of any commercial or financial relationships that could be construed as a potential conflict of interest.

## Publisher's Note

All claims expressed in this article are solely those of the authors and do not necessarily represent those of their affiliated organizations, or those of the publisher, the editors and the reviewers. Any product that may be evaluated in this article, or claim that may be made by its manufacturer, is not guaranteed or endorsed by the publisher.
