# Supplementary figures and images for: Harmonization of Multiple SARS-CoV-2 Reference Materials Using the WHO IS (NIBSC 20/136): Results and Implications
Source: Front Microbiol. 2022 May 30;13:893801. doi: 10.3389/fmicb.2022.893801 (PMC9190986; doi:10.3389/fmicb.2022.893801)

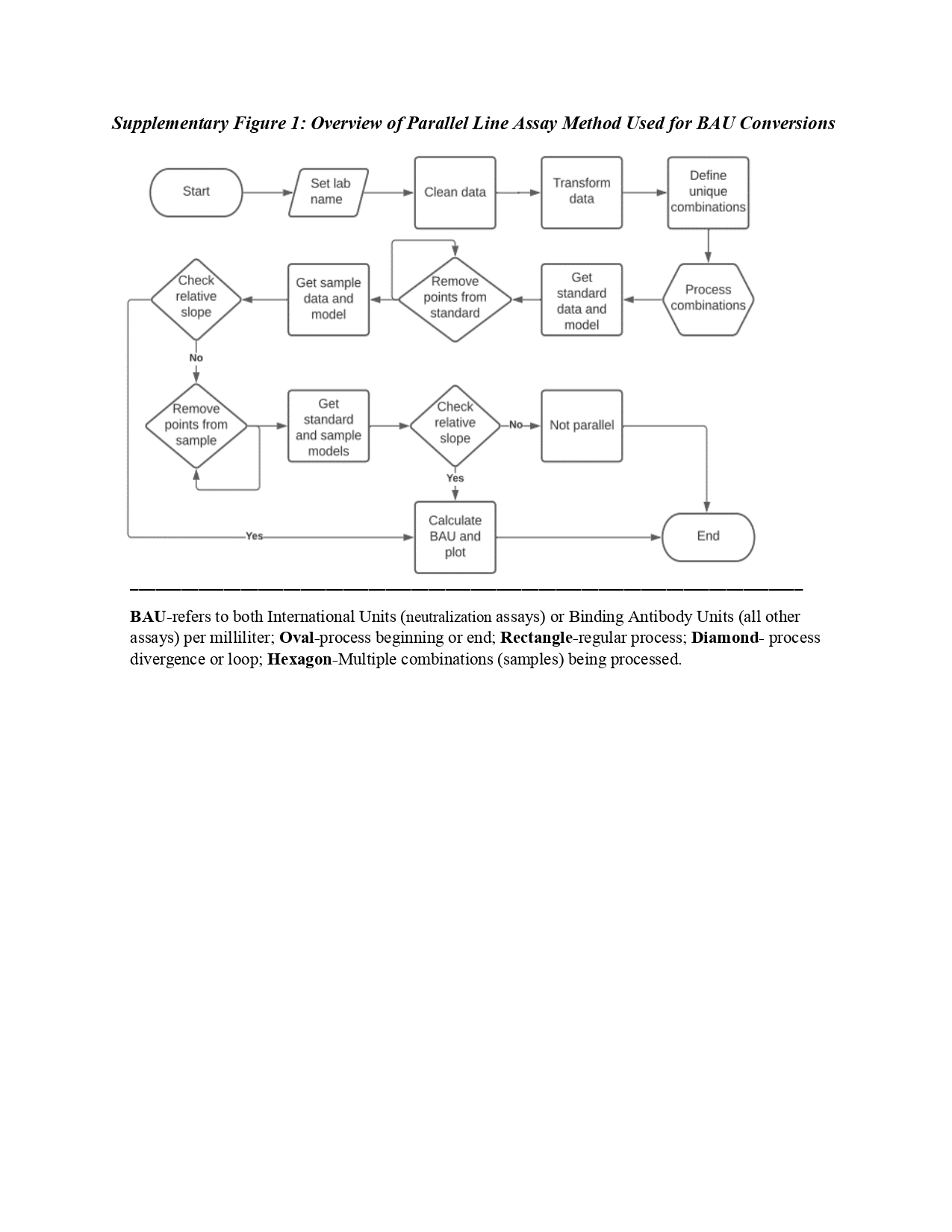

Supplement: Supplementary file 1 [file Data_Sheet_1.zip › Supplementary Figure 1.JPEG]

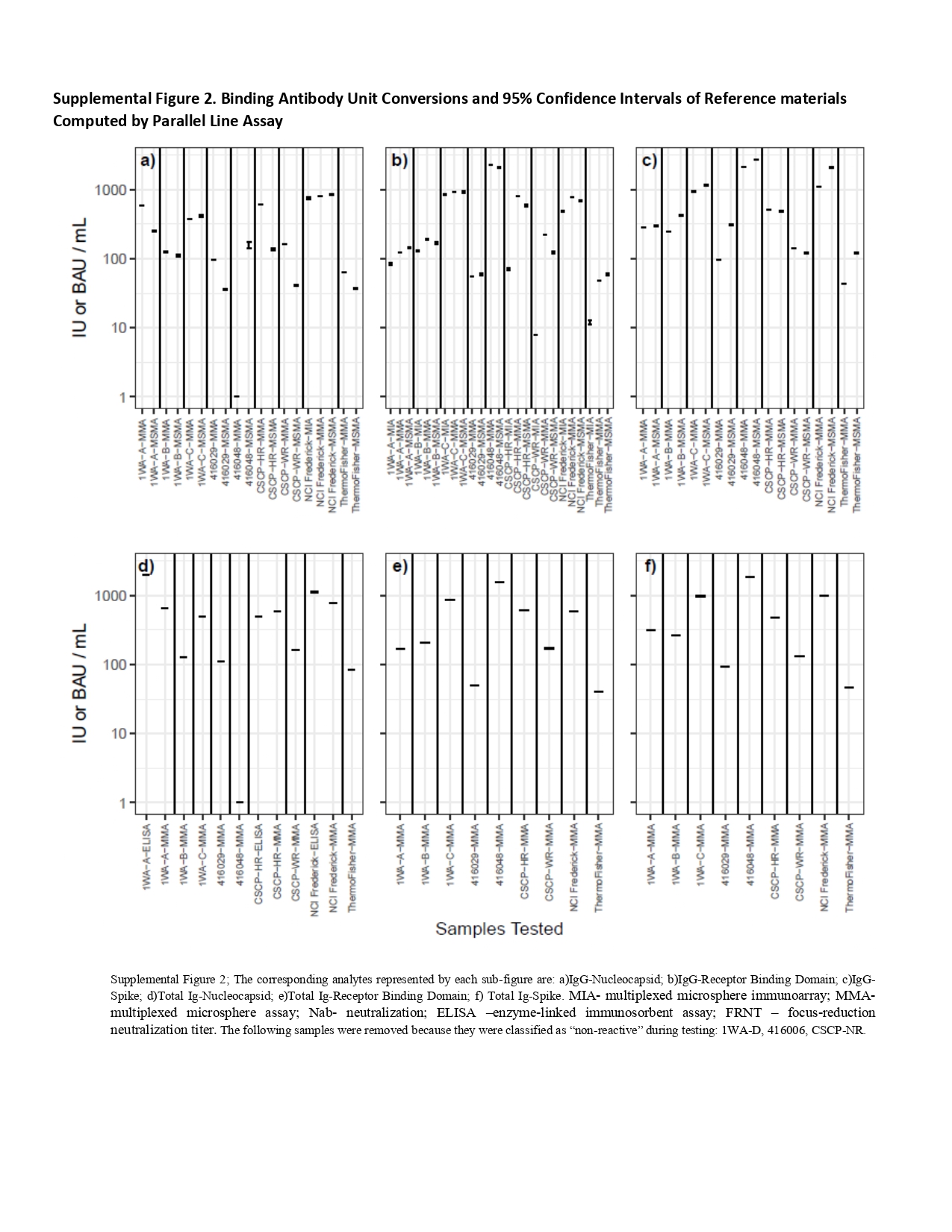

Supplement: Supplementary file 1 [file Data_Sheet_1.zip › Supplementary Figure 2.JPEG]

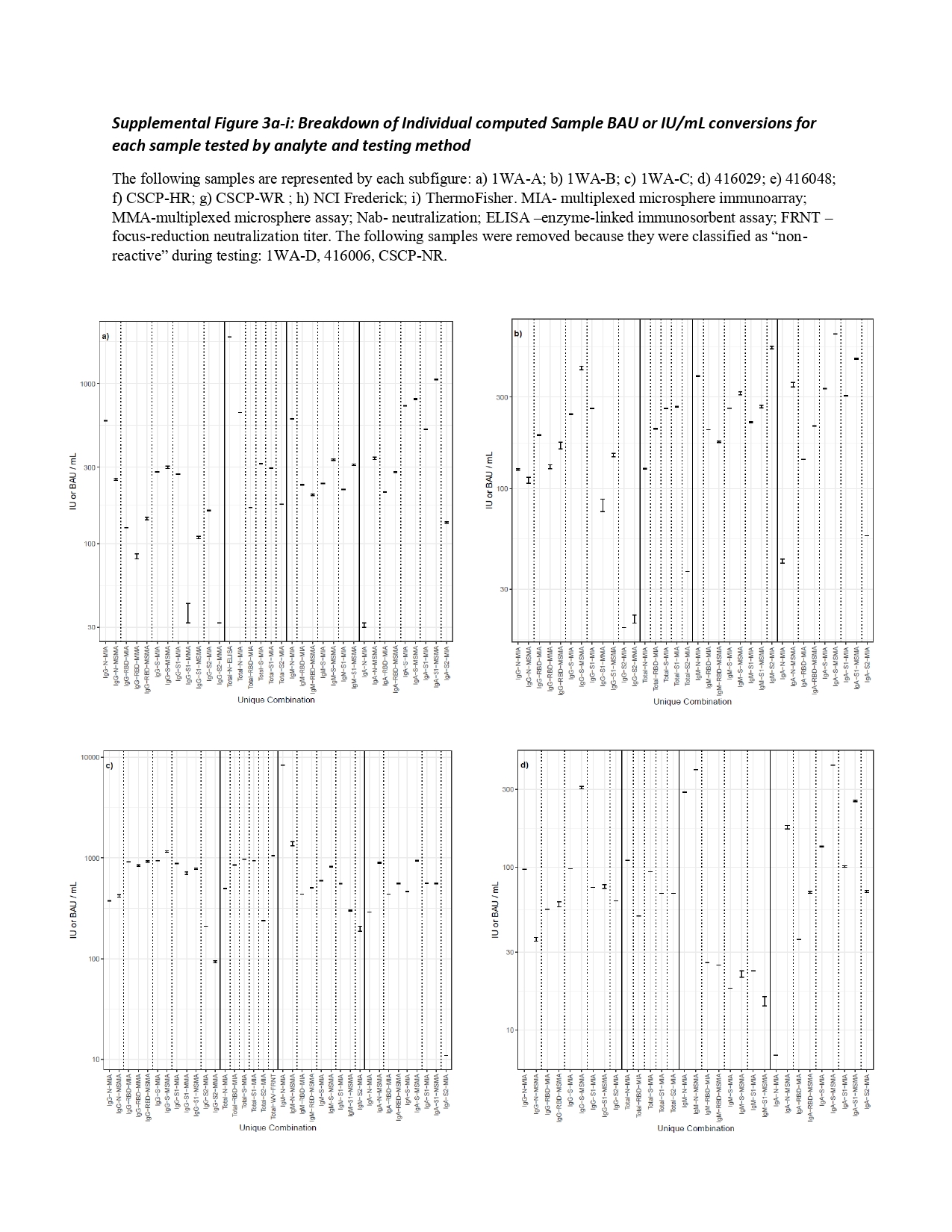

Supplement: Supplementary file 1 [file Data_Sheet_1.zip › Supplementary Figure 3 a-d.JPEG]

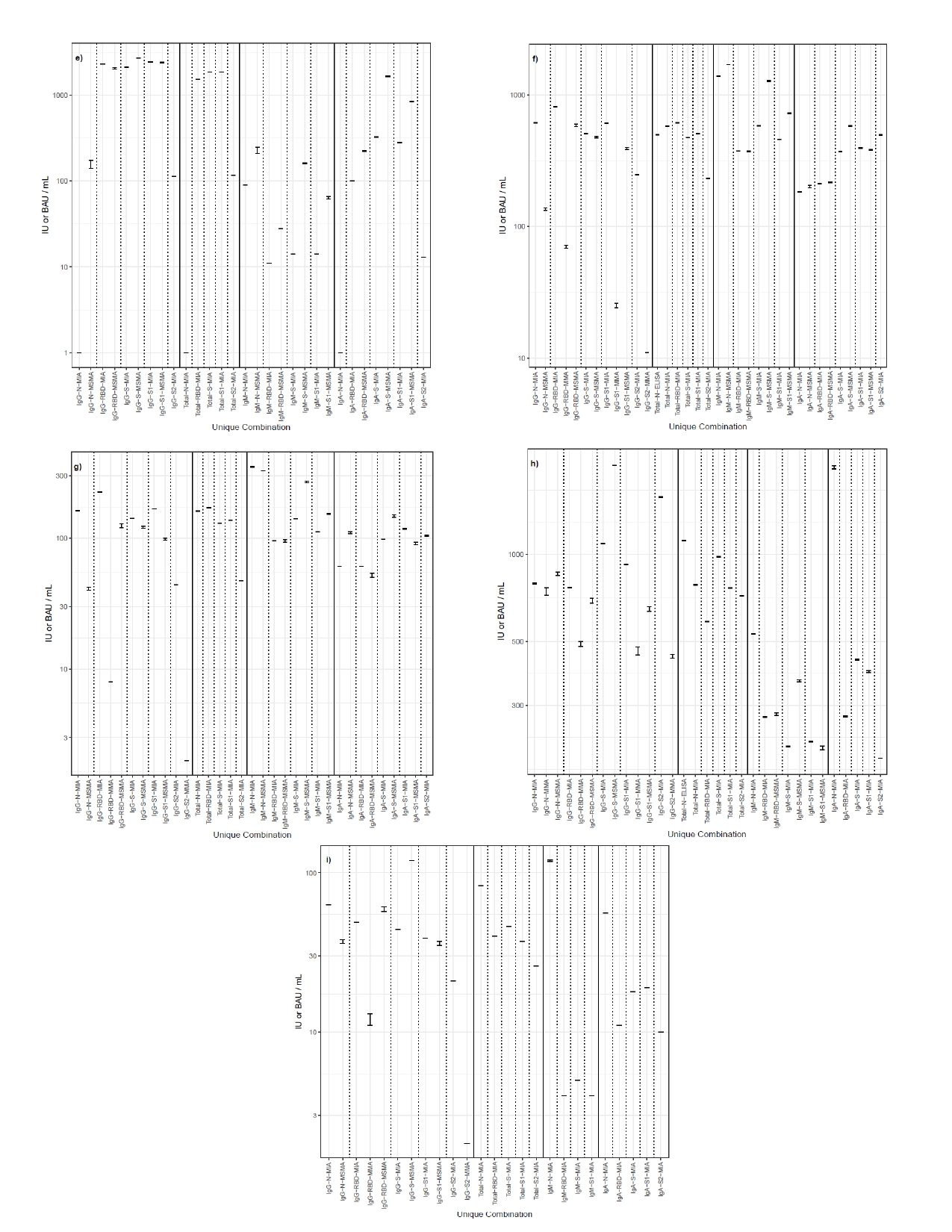

Supplement: Supplementary file 1 [file Data_Sheet_1.zip › Supplementary Figure 3 e-i.JPEG]
